# An open label, randomised controlled trial of rifapentine versus rifampicin based short course regimens for the treatment of latent tuberculosis in England: the HALT LTBI pilot study

**DOI:** 10.1186/s12879-021-05766-9

**Published:** 2021-01-21

**Authors:** J. Surey, H. R. Stagg, T. A. Yates, M. Lipman, P. J. White, A. Charlett, L. Muñoz, L. Gosce, M. X. Rangaka, M. Francis, V. Hack, H. Kunst, I. Abubakar

**Affiliations:** 1 Institute for Global Health, University College London, London, UK; 2 Faculty of Medicine, Universidad Autónoma Madrid, Madrid, Spain; 3 Usher Institute, University of Edinburgh, Edinburgh, UK; 4 Department of Infectious Disease, Faculty of Medicine, Imperial College London, London, W2 1NY UK; 5 UCL-TB and UCL Respiratory, UCL, London, Royal Free London National Health Service Foundation Trust, London, UK; 6 National Infection Service, Public Health, England, UK; 7 Department of Infectious Disease Epidemiology, Imperial College School of Public Health, MRC Centre for Global Infectious Disease Analysis and NIHR Health Protection Research Unit in Modelling Methodology, London, UK; 8 Clinical Sciences Department. School of Medicine, University of Barcelona and Internal Medicine Department, Parc Sanitari Sant Joan de Déu. Sant Boi, Barcelona, Spain; 9 Blizard Institute, Queen Mary University of London, London, UK; 10 Department of Respiratory Medicine, Barts Health NHS Trust, London, UK

**Keywords:** Tuberculosis, Latent tuberculosis treatment, Randomised controlled trial, Rifapentine

## Abstract

**Background:**

Ending the global tuberculosis (TB) epidemic requires a focus on treating individuals with latent TB infection (LTBI) to prevent future cases. Promising trials of shorter regimens have shown them to be effective as preventative TB treatment, however there is a paucity of data on self-administered treatment completion rates. This pilot trial assessed treatment completion, adherence, safety and the feasibility of treating LTBI in the UK using a weekly rifapentine and isoniazid regimen versus daily rifampicin and isoniazid, both self-administered for 12 weeks.

**Methods:**

An open label, randomised, multi-site pilot trial was conducted in London, UK, between March 2015 and January 2017. Adults between 16 and 65 years with LTBI at two TB clinics who were eligible for and agreed to preventative therapy were consented and randomised 1:1 to receive either a weekly combination of rifapentine/isoniazid (‘intervention’) or a daily combination of rifampicin/isoniazid (‘standard’), with both regimens taken for twelve weeks; treatment was self-administered in both arms. The primary outcome, completion of treatment, was self-reported, defined as taking more than 90% of prescribed doses and corroborated by pill counts and urine testing. Adverse events were recorded.

**Results:**

Fifty-two patients were successfully enrolled. In the intervention arm 21 of 27 patients completed treatment (77.8, 95% confidence interval [CI] 57.7–91.4), compared with 19 of 25 (76.0%, CI 54.9–90.6) in the standard of care arm. There was a similar adverse effect profile between the two arms.

**Conclusion:**

In this pilot trial, treatment completion was comparable between the weekly rifapentine/isoniazid and the daily rifampicin/isoniazid regimens. Additionally, the adverse event profile was similar between the two arms. We conclude that it is safe and feasible to undertake a fully powered trial to determine whether self-administered weekly treatment is superior/non-inferior compared to current treatment.

**Trial registration:**

The trial was funded by the NIHR, UK and registered with ISRCTN (26/02/2013-No.04379941).

**Supplementary Information:**

The online version contains supplementary material available at 10.1186/s12879-021-05766-9.

## Keypoints

This pilot trial assessed treatment completion and the feasibility of treating LTBI in the UK using a weekly rifapentine and isoniazid regimen versus daily rifampicin and isoniazid, both self-administered for twelve weeks. Treatment completion was comparable between the two regimens.

## Background

Despite the availability of effective treatment for drug susceptible tuberculosis (TB), it is estimated that a total of 1.5 million individuals died of the disease worldwide in 2018 [1]. In the UK, the majority of TB occurs in migrants, and arises largely through the reactivation of infection acquired overseas [2]. Screening for latent tuberculosis infection (LTBI) is thus an essential element of any TB elimination strategy [3].

In 2011, Sterling et al. described a 12-dose weekly regimen of rifapentine/isoniazid (3HP) with a relatively low likelihood of drug-related hepatotoxicity (0.4%) [4]. This regimen was shown to be non-inferior at preventing the development of active TB to nine months of isoniazid in a randomised controlled trial (RCT). Within that trial, adherence could not be evaluated as patients placed on the 3HP arm had treatment with directly observed therapy (DOT), but those in the control arm did not. Furthermore, one might expect higher rates of completion with a shorter 12-dose regimen of 3HP than nine months of treatment.

In light of the promising results from that RCT [4] and the absence of RCTs comparing 3HP to the UK standard of care of daily rifampicin/isoniazid for three months (3HR), a randomised pilot trial between the two regimens was designed with self-reported treatment completion as the primary outcome. We enrolled adults aged between 16 and 65 years and did not use DOT (treatment was self-administered). Secondary outcomes sought to assess adherence to the regimens using an adherence tool (MARS), as well as the frequency with which adverse events (AEs) were observed in the two study arms. The results of this trial aim to inform the feasibility of a larger, fully powered, trial assessing the non-inferiority of treatment completion on 3HP compared to 3HR.

## Methods

### Trial design and inclusion criteria

Individuals were recruited from two TB clinics in London, UK, between March 2015 and January 2017. Inclusion criteria included age (between 16 and 65 years); LTBI diagnosis by means of an Interferon Gamma Release Assay (QuantiFERON®-TB Gold In-Tube or T-SPOT®.TB) or Tuberculin Skin Test (threshold > 5 mm irrespective of BCG vaccine status); agreeing to accept preventive treatment; and provision of informed consent.

We excluded pregnant and breast-feeding women; persons weighing less than 45 kg;; and individuals unable to receive study drugs due to allergy, liver disease or any medical condition contraindicating the use of a rifamycin or isoniazid; individuals who needed concomitant medications that could not be safely taken with study drugs and those with HIV infection. For this pilot study, we were concerned that patients on antiretroviral therapy might have to have their medication changed if there were randomised to the rifampicin arm due to the drug-drug interaction.

DOT is not standard of care for LTBI treatment in the UK. Individuals whose social circumstances would necessitate enhanced adherence support and DOT - such as homelessness, history of mental health problems, incarceration, or problematic drug use - were also excluded.

After enrolment, individuals were randomised centrally using Sealed Envelope’s web-based Simple Randomisation Service by a member of the study team in the recruitment clinic. There was no stratification and the ratio of individuals in the intervention to standard care arm was 1:1. Recruited individuals were randomised to receive either 3HP (weekly) in the intervention arm or 3HR (daily) in the standard of care arm. Dosing was weight dependant with patients being weighed at each visit and dosing adjusted accordingly (see Additional File 1, Appendix 1 for dosing schedule). Both arms self-administered treatment without direct observation and recruits were educated on how to take the medication as well as the main side effects of treatment.

Treatment was initiated following an eligibility assessment at baseline (week 0). Subsequent medication was dispensed at clinic visits at weeks 2, 4 and 8 following an adherence and AE check as per the study flow chart below. Extra visits were possible if a patient presented with any symptoms related to the study medication or had a mild derangement of liver function tests (see below in AEs).

### Outcomes assessments

#### Primary outcome: treatment completion

The primary outcome was the proportion completing treatment in each arm of the study assessed using self-report. Treatment completion was defined as taking more than 90% of the prescribed doses of treatment. This proportion equates to at least 11 doses for patients taking the weekly intervention regimen and at least 81 doses for patients in the daily standard of care arm. If a patient missed a scheduled appointment, the investigation team tried to contact them within the following week and so total completion had to occur within 16 weeks. Additionally, if participants failed to attend two consecutive appointments, they were considered non-adherent and to have reached one of the endpoints of the study.

Adherence data were collected at regular dispensing clinic visits on weeks 2, 4, 8 and at the end of treatment, using a standardised questionnaire (see Additional File 1, Appendix 2). Adherence and treatment completion was self-reported and assessed by face-to-face enquiry, wherein patients were asked if they had missed any doses of medication since their last clinic visit and the number of tablets reportedly missed was recorded.

We asked participants to bring with them, at each visit, their empty pill packages, which were compared to the number of pills dispensed at the previous visit. At each clinic visit, urine colorimetric testing for detecting isoniazid metabolites and pill count assessment were used to validate the self-reported intake of tablets. The urine tests used a commercially available assay (Isoscreen, GFC Diagnostics Ltd., Oxfordshire, UK). For the purpose of ascertaining treatment completion, we intended to use urinary metabolite and pill count assessment to validate self-reported intake of tablets with either a negative urine test and/or pills still in a pack taken as evidence of non-compliance. Otherwise, self-reports were considered sufficient evidence of outcome.

### Secondary outcomes

#### Mars

A validated medication adherence report scale (MARS™) was used as an assessment of adherence to explore is utility in TB adherence trials. This uses five dimensions: ‘I alter the dose’, ‘I forget to use it’, ‘I stop taking it for a while’, ‘I decide to miss out on a dose’, and ‘I take less than instructed’, using a five-point Likert scale of ‘always’, ‘often’, ‘sometimes’, ‘rarely’ and ‘never’, scored one to five, respectively. An overall score of 20 points or higher is considered high adherence [5, 6]. (See Additional File 1, Appendix 3 for MARS™ questionnaire.)

#### Adverse events

Assessment of AEs for both arms was carried out at clinic visits at weeks 2, 4, 8 and at the end of treatment at week 12. A final post treatment telephone call was made 4 weeks after the end of treatment to check that there had been no further AEs following the last dose of study medication. AEs were assessed from laboratory tests, including regular liver function tests (LFTs) for hepatotoxicity, or from a standardised interview to assess symptoms. They were recorded and graded (1 to 4) according to the Division of AIDS (DAIDS) criteria [7]. The probability of whether the study regimen was related to any AE was assessed by the prescribing physician. Following events scoring 3 or more, medication was stopped; following other AEs, trial medication could be continued at the discretion of the trial physician. For full details of AEs and discontinuation rules, see Additional File 1, Appendix 4.

#### Data collection and statistical analysis

Pseudonymised data were collected on case report forms (CRFs) at study sites. Data were independently double entered at the study co-ordinating centre into a password protected Microsoft Access database. These data were cross validated and corrections made as required to ensure data accuracy. Consistency and error checking was carried out to corroborate the two datasets. An assessment of the distribution of baseline characteristics between the two study arms was used to determine if adjustment for confounding was required. Treatment completion and AEs were described.

## Results

**Fig. 1:**
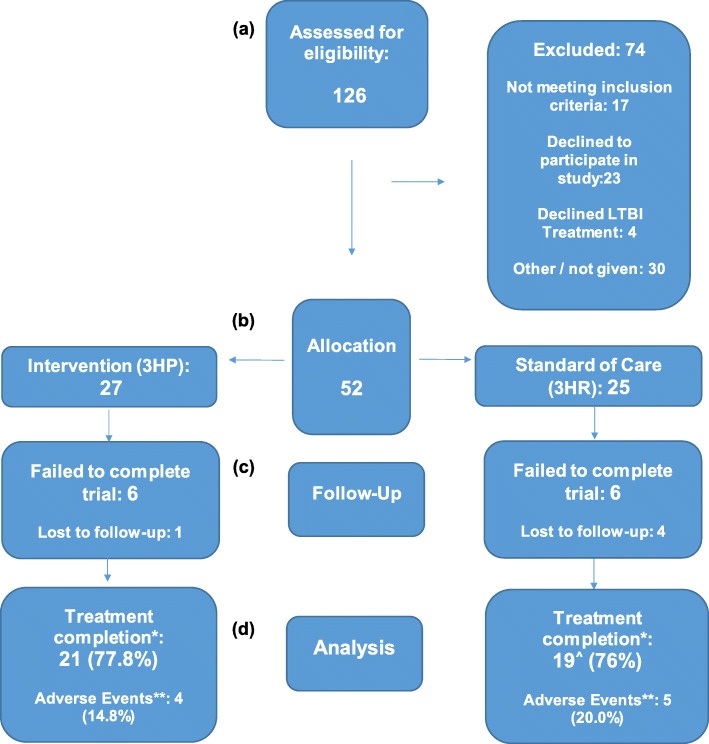
Flow diagram of recruitment of individuals to trial, allocation, and outcomes. **a**: Individuals with latent tuberculosis infection (LTBI) were recruited from TB clinics who agreed to treatment and met the eligibility criteria. **b**: Subjects randomised to weekly rifapentine/isoniazid (3HP) or daily rifampicin/isoniazid (3HR). **c**: Follow-up at 2, 4, 8, 12 weeks. **d**: Treatment completion defined as more than 90% of prescribed doses taken post treatment completion one subject was LFU and another an investigator withdrawal. ** *n* (%) individuals, per arm, that experienced any AE likely due to drug, as per investigator decision

A total of 126 eligible subjects were screened for eligibility and 52 enrolled into the study between March 2015 and January 2017. Of these, 27 (51.9%) subjects were randomly allocated to the intervention treatment arm and 25 (48.1%) to the standard treatment arm (see Fig. 1 for recruitment detail).

A comparison of the baseline characteristics of the two study arms revealed no evidence of major imbalance (See Table 1).

**Table 1:** Comparison of intervention and standard of care arms for potentially important confounding variables at baseline

Variable	Intervention (***n*** = 27)	Standard (***n*** = 25)
Demographic		
Male (%)	13 (48.2%)	13 (52%)
Age: Mean (range)	38.2 (23–56)	32.5 (17–58)
UK Born	2 (7.4%)	3 (12%)
Lifestyle		
Concomitant medication	9 (33.3%)	5 (20%)
Alcohol - current	9 (33.3%)	9 (36%)
Smoking		
Current	3 (11.1%)	6 (24.0%)
Ex	2 (7.4%)	3 (12.0%)
Never	22 (88.5%)	16 (64.0%)
Clinical		
IGRA positive	24 (88.9%)	24 (96%)
TST positive	3 (11.1%)	1 (4%)
Diabetes	1 (3.7%)	1 (4%)
Immunosuppressant medication	1 (3.7%)	–

There were no important differences between the two arms with respect to baseline biochemistry, haematology or clinical signs measurements.

### Primary outcome

Completion of treatment (defined in the protocol as more than 90% of doses taken by self-report) was similar in both arms. In the intervention 3HP arm 21/27 completed compared with 19/25 in the standard of care arm of daily 3HR (See Table 2). Considering the lack of evidence of imbalances between the trial arms, we did not adjust for confounding.

**Table 2:** Counts of self-reported adherence and end of trial completion for intervention and standard drug regimen

Metric	Intervention 3HP *n* (proportion) [CI*]	Standard 3HR *n* (proportion) [CI*]
Allocated to group	27	25
Received at least 90% of prescribed doses	21 (0.78) [0.58–0.91]	19 (0.76) [0.55–0.91]

*CI 95% confidence interval

In both arms there was no clinical appointment where individuals who reported full adherence had pills left and a negative urine test. There were four individuals, two in each arm, who reported full adherence and had pills remaining, but all had positive urine tests indicating they had at least taken some of their prescribed medication. In the standard of care arm, two individuals reported full adherence, had no pills remaining, but had negative urine tests. However, there was a marked discrepancy between the urine test and self-reported full adherence in the intervention arm. In this group 14 (66.7%) individuals had a negative urine test (see Additional File 1, Appendix 5 for detail). We therefore concluded that the gap between pill intake and testing was the most likely explanation and this information was not used to discount self-reported treatment completion.

### Secondary outcomes

#### Adherence using MARS™ tool

There were only two MARS™ ratings of 19, i.e. below the score considered to represent high compliance, one in the standard treatment arm at week two, and one in the intervention treatment arm at week four.

#### Adverse events

There were 122 AEs reported during the trial. There were no serious AEs, as defined by DAIDS criteria as grade 3 or more, recorded during the trial in either study arm. The relationship to the study drugs was considered probable for 25 (20.5%), possibly for 53 (43.4%), unlikely for 35 (28.7%), not related for seven (5.7%) events and a further two which were not attributed a relationship. The 25 AEs that were probably the result of the study drugs occurred in nine subjects and are documented by study arm in Table 3 and further details can be seen in Additional File 1, Appendix 6. No participant developed active TB during the trial.

**Table 3:** Subjects experiencing at least one adverse event likely due to study treatment, by study arm

Experienced an adverse event	Randomised Group		
	Intervention	Standard	Total
No	23	20	43
Yes	4 (14.8%)	5 (20.0%)	9
Total	27	25	52

#### Blood testing and hepatoxicity

Haematological and biochemical tests were performed on subjects throughout the study, with a consideration as to whether these are outside of the normal range. A small number of subjects were observed to fall outside of the normal range for these parameters. Of note are those with raised ALT and AST during the study. There were seven subjects- three in the experimental treatment arm and four in the standard treatment arm that had clinically significant raised ALT results during the trial. In the experimental treatment arm, one subject had raised ALT values throughout the trial; the other two had raised ALTs at one and two measurement points. In the standard treatment arm, two subjects had raised ALTs at two measurement points, the other two having raised ALTs at just one point. These subjects had similar results for ASTs.

As a result, one participant in the standard treatment arm was withdrawn by the investigator as they had LFTs > 3 times ULN (upper limit of normal) and were symptomatic. The remaining participants we able to continue as their results were within acceptable parameters.

Lessons learned and the data generated are summarised to inform future trials (see Table 4, p.12).

**Table 4:** Lessons learned and recommendations of future trials

Issue	Description	Recommendation
Maximising subject recruitment	Out of individuals who were eligible (126) many (23) declined to take part in the study. Anecdotally, this was often because they wanted to receive treatment ‘as normal’. Much of this may be out of the desire to reduce the number of times to attend clinic or perceptions that they were getting something less efficacious or safe. Individuals were required to attend at weeks 0/2/4/8/12 and have a telephone consult at week 16	Simplified patient information sheet (PIS) with a plain English summary Minimise additional follow-up appointments, so care is as close to the standard of care as possible Making sure that all recruiting clinicians are well trained and motivated to recruit and there are dedicated recruitment nurses in clinics Qualitative interviews of perceptions of LTBI treatment and of taking part in clinical trials Flexible times to attend clinics outside of normal working hours
Loss to follow-up (LFU)	6 people were LFU and did not complete the trial. Several were at the end of treatment and so may well have completed treatment but did not want to attend their last appointment.	Larger incentive at end of treatment Qualitative interviews for those LFU
Adherence checks	The isoniazid metabolite urine test frequently came back as negative because participants in the weekly dosing arm had taken the tablets more than 24/48 h before being tested. Participants often took the medication at a time convenient for them e.g. when they were not working or at weekends.	Electronic measures of adherence may be more useful, especially when using a weekly regimen e.g. MEMS cap.
Inclusion criteria	Initially, inclusion criteria specified a positive IGRA test and did not include TST. The protocol was subsequently amended to include both. Individuals under-served populations with social risk factors for poor adherence were excluded	As broad as possible to include anyone eligible to have LTBI treatment in usual clinical practice. Include under-served populations to generate adherence data regarding their adherence.

## Discussion

This is the first UK based RCT comparing the adherence of weekly 12-dose 3HP regimen to the UK standard of care of daily rifampicin/isoniazid for three months (3HR). Within our open label, randomised, multi-site pilot study of the two regimens for LTBI, we found that treatment completion, defined as taking more than 90% of prescribed doses, was comparable between self-administered weekly rifapentine/isoniazid regimen and the UK standard of care regimen of self-administered daily rifampicin/isoniazid. Furthermore, the frequency and severity of the AEs of the two regimens was similar.

Previous research has demonstrated that self-administered 3HP is non-inferior to directly observed 3HP [8]. Treatment completion rates of 3HP are high when administered using DOT and non-inferior when compared with longer duration regimens such as isoniazid monotherapy over nine months [4, 9, 10]. In this pilot study, we have shown that a fully powered trial efficacy trial comparing these two self-administered regimens is possible, allowing a determination of whether the 12 weekly 3HP is superior/non-inferior the UK standard of care.

This pilot used self report as the primary measure of adherence with an adherence tool, MARS™, as a secondary measure. We had hypothesised based on evidence that it works in asthma and other chronic conditions and that if shown to be a good measure of outcomes in TB, it can provide an alternative outcome measure in future trials. Unfortunately, it was not very discriminating as almost everyone had a high score in the pilot study. However, we also feel, despite the absence of effect in our relatively small study, that this merits further exploration in the full trial.

Lessons learned and the data generated in this trial have contributed to the design of the RID-TB trial [11] which will be fully powered to estimate the likelihood of treatment completion of the two regimens and the relative safety and efficacy of daily 3HR as compared with weekly 3HP. Subject to cost-effectiveness analyses, weekly 3HP regimens could be a useful option in preventative therapy, especially to improve coverage of populations who may require adherence support. People living with HIV are eligible to take part in the full trial. However, concurrent medication will be carefully reviewed to ensure that individuals in need for treatment that cannot be safely taken together with study drugs will be excluded.

However, caution is needed in interpreting these results as this was a pilot study which was not powered to test equivalence or non-inferiority for any outcome. The size of the trial means that we were unable to assess the efficacy of the intervention. In this study we excluded people with risk factors for potential poor treatment adherence, such as homelessness, problematic drug use, mental health concerns or history of incarceration. Therefore, our results will not be generalised to these populations. One advantage of a weekly regimen is that it could make treating these patient groups more feasible as ensuring adherence once a week is less resource intensive than every day.

Finally, treatment completion was defined as completing 90% of doses taken within 125% of the timeframe of a normal course of treatment. The simplistic nature of a percentage threshold may mask substantial heterogeneity that could lead to different therapeutic coverages in different patients. There is little good data regards how adherence patterns - which can be highly complex [12] - relate to outcomes and exactly what level of adherence is optimal for any given regimen is unclear [13–15].

In considering the fully powered trial, other short regimens that have been shown to be effective since the start of the HALT trial should be considered. For example, a large trial amongst HIV infected individuals, in an area with high TB prevalence, showed that a one-month daily rifapentine plus isoniazid (1HP) daily regimen was non-inferior to 9 months of isoniazid alone in preventing active TB and had fewer AEs. Whilst patients self-administered treatment under trial conditions, 97% of those who started 1HP treatment completed it, according to self-reported data [16]. This large trial also had a major limitation due to the potential enrolment of individuals who may not have LTBI [17] suggesting that further trial efficacy data may be needed prior to an adherence study of 1HP, especially in the light of the recommendation of expanding 1HP regimens [18].

## Conclusion

In the first RCT of its kind in the UK, we demonstrate similar treatment completion rates between a weekly 3HP regimen and the daily 3HR standard of care. We have also demonstrated the feasibility of undertaking a randomised controlled trial of the two regimens, powered to estimate the relative efficacy of the two regimens.

## Supplementary Information

**Additional file 1: Appendix 1**: Dosing schedule. **Appendix 2**: Standardised questionnaire. **Appendix 3**: MARS™ Adherence Questionnaire. **Appendix 4**: Adverse events and discontinuation rules. **Appendix 5**: Comparison of self-reported adherence and isoniazid urine test. **Appendix 6**: Adverse events

**Additional file 2:.** CONSORT 2010 checklist of information to include when reporting a randomised trial

## Data Availability

The datasets used and/or analysed during the current study are available from the corresponding author on reasonable request. The protocol can also be provided on request.

## Abbreviations

AE: Adverse events
BCG: Bacillus Calmette–Guérin
CI: Confidence interval
CRF: Case Report Form
DAIDS: Division of AIDS
DOT: Directly observed therapy
HALT: Hepatitis and Latent Tuberculosis study
HP: Isoniazid Rifapentine
HR: Isoniazid Rifampicin
IGRA: Interferon Gamma
LFTs: Liver function tests
LFU: Lost to follow-up
LTBI: Latent tuberculosis infection
MARS: Medication adherence report scale
NHS: National Health Service
PIS: Patient Information Sheet
RCT: Randomised controlled trial
TB: Tuberculosis
