# Supplementary material for: An open label, randomised controlled trial of rifapentine versus rifampicin based short course regimens for the treatment of latent tuberculosis in England: the HALT LTBI pilot study
Source: BMC Infect Dis. 2021 Jan 21;21:90. doi: 10.1186/s12879-021-05766-9 (PMC7818935; doi:10.1186/s12879-021-05766-9)
Supplement: Supplementary file 1 — Additional file 1: Appendix 1: Dosing schedule. Appendix 2: Standardised questionnaire. Appendix 3: MARS™ Adherence Questionnaire. Appendix 4: Adverse events and discontinuation rules. Appendix 5: Comparison of self-reported adherence and isoniazid urine test. Appendix 6: Adverse events [file 12879_2021_5766_MOESM1_ESM.docx]

**Appendix 1**

**Dosing schedule**

| **Study Arm** | **Interventions** |
| --- | --- |
| Standard care arm:  Rifampicin/Isoniazid daily (3HR) | Agent: Rifinah^®^ (rifampicin and isoniazid) for 90 days (3 months).  Daily dosage per weight at baseline or last visit:   \| 50 kg or less \| above 50 kg \| \| --- \| --- \| \| 3 x Rifinah^®^ 150/100 \| 2 x Rifinah^®^ 300/150 \|   All received Pyridoxine 10 mg or 25 mg* with their dose (once daily for 90 days) |
| Intervention arm:  Rifapentine/isoniazid weekly (3HP) | Agents: Priftin^®^ (rifapentine) plus isoniazid for 12 weeks (12 doses)  Weekly dosage per weight at baseline or last visit:   \| < 50 kg \| ≥ 50 kg \| \| --- \| --- \| \| 5 x Priftin^®^ 150 mg  (Rifapentine 750 mg)  +  Isoniazid 15 mg/kg  300 mg and or 150 mg oral tablets used, therefore:  45-50 kg = 750 mg isoniazid (3 pills) \| 6 x Priftin^®^ 150 mg  (Rifapentine 900 mg)  +  Isoniazid 15 mg/kg  300 mg and or 150 mg oral tablets used, therefore:  50-55 kg = 750 mg isoniazid (3 pills)  >55 kg = 900 mg isoniazid (3 pills) \|   All received Pyridoxine 10 mg or 25 mg with their dose (once weekly for 12 weeks)* |

*as per local practice

**Appendix 2**

**Standardised questionnaire**

| **1.** | **COMPLIANCE** | | |
| --- | --- | --- | --- |
|  | **Did you forget one or more doses? If you did, how many?** | ☐ No | ☐ Yes  Number: __ |
|  | **Urine testing** | ☐ Negative | ☐ Positive |
|  | **Pill counts: Count the number of remaining pills** |  | Number of pills given in last visit: __ |
|  | **MARS-5 test (see below)** | **Score:** | |
| **2.** | **ADVERSE EVENTS** | | |
|  | **Have there been any Adverse Events?**  (If yes, please record in Adverse Events Log) | ☐ No | ☐ Yes |
|  | **Did the patient self report any symptoms?**  **(If yes, please record in Adverse Events Log)** | ☐ No | ☐ Yes (specify) |
|  | **Did the patient have any of these symptoms:**   - jaundice - nausea - vomiting - abdominal pain - fever - rash   **(If yes, please record in Adverse Events Log)** | ☐ No  ☐ No  ☐ No  ☐ No  ☐ No  ☐ No | ☐ Yes  ☐ Yes  ☐ Yes  ☐ Yes  ☐ Yes  ☐ Yes |
| **3.** | **OTHER CONDITIONS** | | |
|  | **Has the Has the patient had any health problems since the last visit?** (i.e. visit to GP or A&E)  **Have there been any changes in Concomitant Medications?**  (If yes, please record in Concomitant Medications Log | ☐ No  ☐ No | ☐ Yes  ☐ Yes |

**Appendix 3**

**MARS^TM^ Adherence Questionnaire**

**Patient Study ID**:◻-◻◻◻

**Treatment phase: Week Number** ___


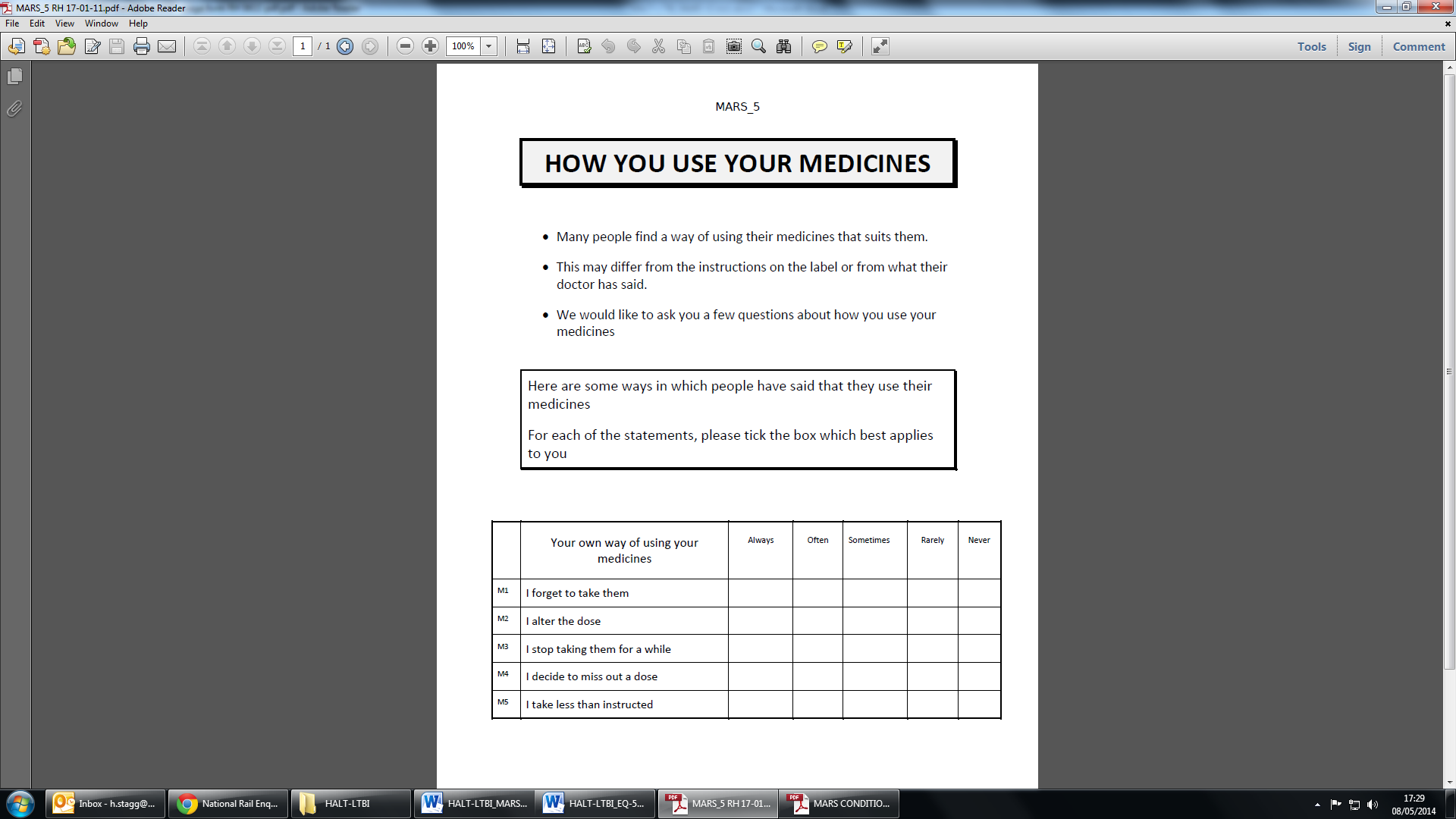


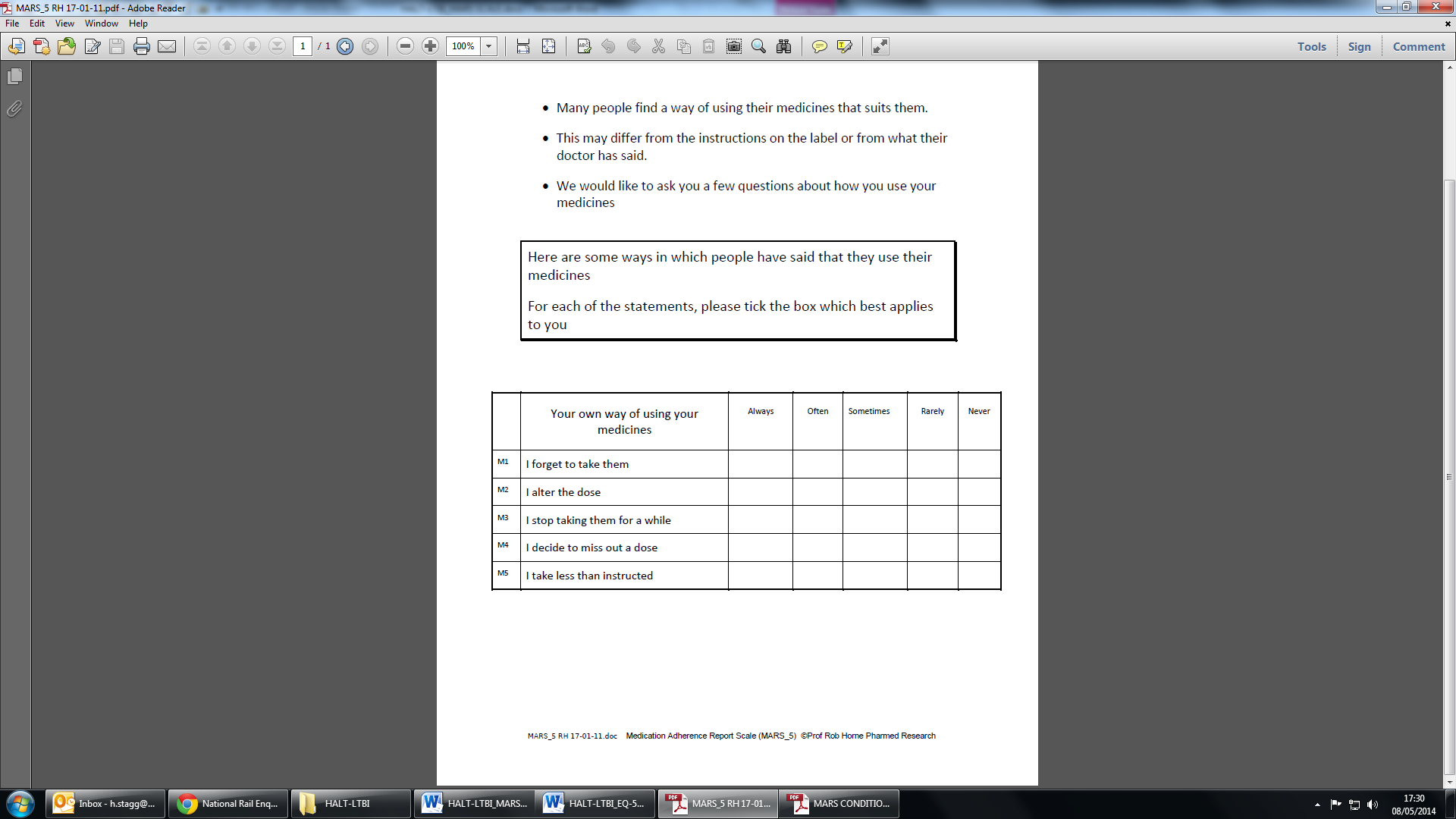


**Appendix 4**

**Adverse events and discontinuation rules**

**Adverse events:** AEs were assessed from laboratory tests or from a standardised interview to assess symptoms. They were recorded and graded (1 to 4) according Division of AIDS (DAIDS) criteria [7]. Following events scoring 3 or more, medication was stopped, following other AEs, trial medication could be continued at the direction of the trial physician.

- Laboratory tests
  - Liver function tests (LFTs, consisting of transaminases AST/ALT, total bilirubin and alkaline phosphatase).
  - Full blood count (haemoglobin, haematocrit, platelets and white blood cell differential count),
  - Biochemistry (fasting blood glucose, urea, creatinine, potassium and sodium).
- Standardised interview
  - Symptom enquiry of jaundice, nausea/vomiting, abdominal pain, fever or skin rashes.
  - Individuals about any other heath problem that led them to see their GP, attend a hospital appointment, or hospital admission since they started taking the medication.

Following events scoring 3 or more, medication was stopped, following other AEs, trial medication could be continued at the direction of the trial physician. If in one of the scheduled visit, any LFT derangements or symptoms of hepatitis have been detected, LFTs and other investigations may be repeated at a shorter interval. If ALT or AST are over 5 times the upper limit of normal (ULN) in the absence of symptoms, the patient will stop the treatment. If they have symptoms, the limit of LFT for stopping treatment is 3 times ULN. In other circumstances (LFT under the pre-established limits), patients can safely continue their treatment. They will return to the established visits once the LFT are lowered. If the medication has been stopped, it will not be reintroduced again and the patient will be referred back to routine clinical care.

**Discontinuation/withdrawal of participants and ‘stopping rules’**

- Withdrawal of consent at any stage
- Female participant becoming pregnant.
- Lost to follow up (defined as a patient non-attending the clinic or not willing to accept study procedures for two consecutive visits).
- Subsequent demonstration of isoniazid or rifamycin resistant strain in the index case
- Development of active TB
- Missing x2 consecutive appointments
- Adverse events (see above)

Individuals withdrawn from the trial would be followed up for ongoing treatment and care at the same clinical site.

**Appendix 5**

**Comparison of self-reported adherence and isoniazid urine test:** Proportion of those who reported taking all prescribed medication at clinical appointments and concordance with isoniazid urine test

| **Self-reported full adherence with** | **Intervention**  n=21 (%) | **Standard**  n=19 (%) |
| --- | --- | --- |
| Pills remaining | 2 (9.5) | 2 (10.5) |
| Negative urine test | 14 (66.7) | 2 (10.5) |
| Indeterminate urine tests* | 3 (14.3) | 0 |

*Metabolites are detectable for up 48 hours post dosing, however, this can be affected by the acetylator status of the patient. Indeterminate tests are a partial colour change, as per manufactures instructions.

There was a notable discordance between those who reported reporting taking all prescribed doses and isoniazid urine testing in the weekly intervention arm. Individuals taking the weekly dose, as treatment was self-administered, could have taken medication a number of days prior to any clinic appointment. The use of isoniazid testing in this scenario is therefore questionable (see Table 4 – Lessons Learned)

**Appendix 6**

**Adverse events**

There were no serious AEs reported during the trial in either study arm.

A total of 26 trial subjects reported having AEs during the trial; these are summarised in Table 4. Of these, 15 (55.6%) were in the intervention treatment arm, and 11 (44.0%) in the standard treatment arm.

**Table 4: Number and percentage of subjects experiencing at least one adverse event by study arm**

| **Experienced an adverse event** | **Randomised Group** | | |
| --- | --- | --- | --- |
|  | **Intervention** | **Standard** | **Total** |
| No | 12 | 14 | 26 |
| Yes | 15 (55.6%) | 11 (44.0%) | 26 |
| Total | 27 | 25 | 52 |

Of the 38 subjects that completed the trial, 20 (52.6%) reported at least one AE, compared to 6 (42.9%) of the 14 that withdrew from the trial.

There were a total of 122 AEs reported during the trial. The relationship to the study drugs were considered probably for 25 (20.5%), possibly for 53 (43.4%), unlikely for 35 (28.7%), and not related for 7 (5.7%) of events. Of the two AEs that were not attributed a relationship, in one subject the description ‘felt dizzy after taking rifampicin and isoniazid. Felt better after pyridoxine’, and for the other ‘developed cold and headache. Paracetamol 2x4 times given, [patient] feels better’.

Sixteen (13.2%) of the AEs were considered to be of moderate severity, and 105 of mild severity. For one severity was not recorded. Again, there appeared to be no strong link between such AEs and trial completion, with six (15.8%) of those completing, and three (21.4%) of those withdrawn, reporting AEs probably due to the study treatment.

There were a total of 20 recorded AEs in five subjects which were reported as not resolved. All but one of these subjects completed the trial
